# Heterologous microarray experiments allow the identification of the early events associated with potato tuber cold sweetening

**DOI:** 10.1186/1471-2164-9-176

**Published:** 2008-04-16

**Authors:** Paolo Bagnaresi, Anna Moschella, Ottavio Beretta, Federico Vitulli, Paolo Ranalli, Pierdomenico Perata

**Affiliations:** 1 CRA-GPG, Genomic Research Center, Via S. Protaso 302, I-29017 Fiorenzuola d'Arda (PC), Italy; 2 CRA-CIN, Research Center for Industrial Crops, 40128 Bologna, Italy; 3 Genopolis, Department of Biotechnology and Bioscience, University of Milano-Bicocca, 20126 Milan, Italy; 4 CRA-DTI, Department of Processing and Technology Development of Agricultural Products, Roma, Italy; 5 Plant and Crop Physiology Laboratory, Scuola Superiore Sant'Anna, 56127 Pisa, Italy

## Abstract

**Background:**

Since its discovery more than 100 years ago, potato (*Solanum tuberosum*) tuber cold-induced sweetening (CIS) has been extensively investigated. Several carbohydrate-associated genes would seem to be involved in the process. However, many uncertainties still exist, as the relative contribution of each gene to the process is often unclear, possibly as the consequence of the heterogeneity of experimental systems. Some enzymes associated with CIS, such as β-amylases and invertases, have still to be identified at a sequence level. In addition, little is known about the early events that trigger CIS and on the involvement/association with CIS of genes different from carbohydrate-associated genes. Many of these uncertainties could be resolved by profiling experiments, but no GeneChip is available for the potato, and the production of the potato cDNA spotted array (TIGR) has recently been discontinued. In order to obtain an overall picture of early transcriptional events associated with CIS, we investigated whether the commercially-available tomato Affymetrix GeneChip could be used to identify which potato cold-responsive gene family members should be further studied in detail by Real-Time (RT)-PCR (qPCR).

**Results:**

A tomato-potato Global Match File was generated for the interpretation of various aspects of the heterologous dataset, including the retrieval of best matching potato counterparts and annotation, and the establishment of a core set of highly homologous genes. Several cold-responsive genes were identified, and their expression pattern was studied in detail by qPCR over 26 days. We detected biphasic behaviour of mRNA accumulation for carbohydrate-associated genes and our combined GeneChip-qPCR data identified, at a sequence level, enzymatic activities such as β-amylases and invertases previously reported as being involved in CIS. The GeneChip data also unveiled important processes accompanying CIS, such as the induction of redox- and ethylene-associated genes.

**Conclusion:**

Our Global Match File strategy proved critical for accurately interpretating heterologous datasets, and suggests that similar approaches may be fruitful for other species. Transcript profiling of early events associated with CIS revealed a complex network of events involving sugars, redox and hormone signalling which may be either linked serially or act in parallel. The identification, at a sequence level, of various enzymes long known as having a role in CIS provides molecular tools for further understanding the phenomenon.

## Background

Affymetrix GeneChips have set the standard in terms of reproducibility, sensitivity and other quality parameters, even among other oligonucleotide arrays that are usually the most reputable in terms of reliability compared to other microarray platforms [1,2]. The array design of Affymetrix includes multiple probes for each gene and the presence of mismatch (MM) as well as perfect mach (PM) probes. These features can be exploited in order to minimize major problems in genome-wide approaches such as cross-hybridization, background and noise [3]. Unfortunately, plant-related Affymetrix GeneChips are only available for *Arabidopsis*, sugarcane, tomato, *Vitis vinifera*, soybean, rice, poplar, *Medicago*, cotton, *Citrus*, maize, and barley. One possible solution to this limitation would be to perform unassisted heterologous hybridizations. Although this approach has not been endorsed by Affymetrix itself, it has nonetheless resulted in some valuable achievements, e.g. [4]. This is especially true for the plant kingdom, as the availability of several microarray platforms for *A. thaliana *has been exploited with several studies addressing close relatives of this model species [[5,6] and references therein]. However, most researchers refrain from using heterologous approaches due to the uncertainties in the interpretation of results. To help overcome these problems, one approach recently developed for the Affymetrix GeneChip platform is the NASCarrays Xspecies project, which is based on the preliminary hybridization to the GeneChip of genomic DNA for the species of interest [7,8]. Here we describe the development of an alternative approach, based on the generation of a list (Global Match File) where GeneChip target sequences are aligned to EST-derived clusters/singletons from the species of interest [TIGR Transcript Assemblies, TIGR TA [9]]. This means that: (i) any alignment can be assessed among an available plant GeneChip target sequence and its heterologous counterpart transcript; (ii) an updated and high quality annotation (based on TIGR TA) can be used for this transcript; (iii) a subset of "highly reliable" probesets in the GeneChip species can be generated whose alignments to ESTs of the species of interest are above a threshold and thus can be considered without further substantial scrutiny.

High-throughput sequencing in recent years has produced large amounts of EST data. Based on TIGR plant transcript assemblies (TIGR TA), more than 1,000 ESTs or cDNA sequences are available for 254 plant species reaching a sum of 10,974,563 ESTs (Current Release Summary, update July 2007). Well-known species such as the potato present a total of 81,072 (26,280 TA plus 54,792 singletons) suggesting a fair transcriptome coverage. In cases of low sequence representation, which are typical, for example, of wild relatives of crop species, it can be argued that even transcript profiling of just a few hundred (fully validated) genes in a species of interest would be cost-effective when compared to traditional methods. In fact, while full-reliability of expression data in a heterologous approach would be based on assessing the sequence similarity between ortholog genes of the two species, a much higher amount of crude expression data would be available that would span all genes represented in the GeneChip.

We have chosen the potato as a study organism to query the tomato GeneChip microarray consisting of 10,038 *Solanum lycopersicum *probesets representative of over 9,200 genes. In fact, Affymetrix has not yet developed a potato GeneChip, although spotted cDNA arrays (e.g. TIGR) have been available for the potato for some time. However, TIGR Potato cDNA Microarray distribution has recently been discontinued [10]. Potato and tomato appear to be suitable for conducting a heterologous approach as the two Solanaceous species are strictly phylogenetically related [11]. In fact, several groups have concluded that at least for core genes exhibiting significant sequence homology to the selected solanaceous platform [e.g. potato or tomato spotted cDNA arrays [11,12]] reliable gene expression values can be obtained. Yet it is unclear which genes are eligible for the "core" definition, indicating that a precise validation procedure is required.

Our heterologous approach was conceived as a prerequisite step for investigating a well-known phenomenon, namely the potato tuber cold-induced sweetening, which has been studied for more than 120 years since its first description [13]. Incubating potato tubers at 2–8°C causes the accumulation of sugars (mainly sucrose, glucose and fructose) at the expense of starch, and is therefore detrimental for tuber quality despite delayed tuber sprouting [14]. Furthermore, upon cooking, dark, bitter tasting melanoidins are produced by Maillard reaction involving reducing sugars (as glucose and fructose) and free amino acids[15]. In recent years, there has been increased concern since a specific type of Maillard reaction involving the amino-acid asparagine (abundant in potato tubers) and reducing sugars has been shown to produce the genotoxic and neurotoxic compound acrylamide [15].

Since the discovery of cold sweetening, the various genes playing a key role in the carbohydrate metabolism from starch degradation to sucrose synthesis and breakdown have been investigated [14]. However, the precise contribution of each gene is still unknown and there is no detailed overall picture of the early events triggering cold sweetening. This could be in part attributable to the heterogeneity of the systems used (tuber varieties, experimental settings, etc.) and is an unavoidable drawback of low-throughput expression profiling techniques that focus on one or a few genes that code for well-known enzymes.

Some agreement exists on the following early, cold-induced events as being causative/associated with cold sweetening. At least one amylase activity, demonstrated to be a β-amylase based on substrate specificity, was visible very early in iodine-stained zymograms [3-day cold induction at 3°C; [16,17]]. Sucrose phosphate synthase (SPS) underwent a change in kinetic properties and its transcript was induced in a few days at temperatures of 5–3°C [17]. UDP-glucose-pyrophosphorylase (UGPase) transcript level was high in developing tubers but an increase was also evident upon cold incubation of tubers [18]. Also, specific UGPase isoforms may be associated with increased susceptibility to cold sweetening [14]. Acid invertase was observed as playing a critical role in reducing sugar accumulation and its transcripts were found to be strongly induced after a few days at 4°C; however, the total amount of invertase activity was found to be solely related to the hexose/sucrose ratio rather than total reducing sugars [19].

In this study, we have explored the feasibility of a heterologous GeneChip approach which we believe should be of great interest for researchers dealing with species for which no GeneChip is available. We tested our heterologous approach on potato tuber cold-induced sweetening, a phenomenon known for over a hundred years and which is nowadays extremely important as it has been shown to strongly enhance acrylamide formation in potato processed products.

Apart from investigating heterologous GeneChip approaches, our main aims were: (i) to identify early cold-responsive gene family members to be studied in more detail by qPCR over a 26-d time course; (ii) to obtain an overall and unifying transcriptional picture of these genes, including those previously studied, taking advantage of the use of a single, standardized system (i.e. same genotype and experimental settings); (iii) to obtain a crude, global view of early expression trends for those genes that are not carbohydrate-associated.

## Results and Discussion

### The "Global Match File" as an assisting tool for tomato vs. potato heterologous hybridizations

A Global Match File was generated in order to address the following issues: (i) considering a given tomato probeset, which (if any) potato transcript(s) are reliably represented and what is the quality of this association; (ii) retrieval, for such a potato transcript, of an updated and high quality annotation (based on TIGR TA); and (iii) generation of subsets of "highly reliable" probesets whose alignment scores to potato counterparts are above a tuneable threshold and thus can be considered with confidence. The Global Match File (see additional files 1 and 2) consists of 269,474 alignments (see methods for further details). We have added several indexes for assessing alignment fidelity among tomato and potato sequences. Table 1 shows the most relevant parameters contained in the Global Match File and their intended use. "ALL_90%" and "ALL_70%" sets are grouped into separate sheets and include alignments scoring above the 90% and "70%_PERF_ALIGN" threshold, respectively. As a single probeset can produce more than one hit, both > 90% and > 70% sheets are accompanied by a further sheet that lists non-redundant probesets above the threshold.

**Table 1:** Explanation of parameters contained in the Global Match File.

**Parameter**	**Description**	**Intended use and comments**
TOMATO_AFFY_ID	Tomato Probeset #ID	Tomato Affymetrix probeset identification
POTATO_EST	ID of aligning potato TA/EST	Potato TA/EST identification
DESCRIPTION	TIGR TA annotation	Updated TIGR annotation of potato TA/EST
%_ALIGN/TARG	Ratio between aligned portion of potato TA/EST against the total length of the tomato target (ALIGN_LENGTH/LENGTH_TOM)*100	Rough alignment evaluation between the tomato target and potato TA/EST. Scores higher than 100% reveal gaps in the alignment.
%_PERF_ALIGN	Corrected ratio between aligned portion of potato EST against the tomato target (%_ALIGN/TARG*%_IDENTITY)/100	This improvement of %_ALIGN/TARG parameter is needed to get the percentage of the net alignment, thus hiding the effects of non-contiguous alignment regions. Scores of 100% mean that the entire length of the tomato target sequence is aligned.
%_STOP_DIST	Ratio between the position of the last nucleotide aligned in the tomato target against the total length of the tomato target (LAST_POS_TOM/LENGTH_TOM)*100.	- Similar, lower than 100% values of %_PERF_ALIGN and %_STOP_DIST suggest alignment interruption due to an intervening 3' UTR (outside coding sequence) -Very different values of %_PERF_ALIGN and %_STOP_DIST suggest alignment interruption within coding sequence.

Various indexes were developed in the Global Match File in order to characterize alignment quality between tomato target and matching potato counterparts. The most relevant parameters are listed above along with their intended use in terms of assessing alignment quality.

As the Affymetrix GeneChip target region design procedure [with some exceptions such as *Arabidopsis *ATH1 GeneChip; [20]] is routinely aimed at 3' ends of mRNA, we observed homology drops downstream of the stop codon attributable to lower evolutionary conservation in 3' UTR. Thus, in cases where it is desirable to query low %_PERF_ALIGN values, we developed a further index, "%_STOP_DIST", (Table 1) allowing to assess the possible presence of a stop in the alignment (see examples in Table 1). In some cases, however, small stretches of high homology were further present downstream of the stop codon, and are listed as additional match lines in the Global Match File.

To summarise, the following use of the Global Match File is proposed: a tomato probeset of interest can be simultaneously searched for in all the sheets and thus the best matching potato counterpart and updated annotation can be determined. If the searched probeset matches the > 90% PERF_ALIGN list, this generally indicates that there is a high-confidence match to a potato counterpart. Inclusion in the > 70% PERF_ALIGN still indicates fair sequence similarity, and in most cases this is sufficient for large-scale analysis. For matches slightly below 70% PERF_ALIGN further matches between the same probeset and potato TA should be searched, which may add up to the desired % PERF_ALIGN value. If no further alignments are revealed, then further cases as described in Table I should be investigated to see if they apply (intervening stop). Finally, in cases of even lower % PERF_ALIGN, valuable information can still be obtained by analyzing identity at a probe level (e.g. Affymetrix probe match facility) including .cel files in order to disaggregate probeset signals. In this way, signal intensities associated with perfectly matching probes can be evaluated directly.

Despite these "advanced" procedures to recover specific genes of interest, the 70%_PERF_ALIGN threshold in our dataset was satisfactory as 6,690 out of 10,038 probesets were conserved. In addition, in only rare cases of genes of special interest, further analysis of low %_PERF_ALIGN probesets was necessary. The 70%_PERF_ALIGN threshold thus seems a good compromise between the number of conserved probesets and the fidelity of alignments, especially in cases of alignment drop due to intervening stop codons as found in the tomato GeneChip. In any case, with the higher-confidence threshold of 90%_PERF_ALIGN, 4,893 unique probesets were still conserved.

Our GeneChip dataset reveals 1,854 differentially expressed genes (DEG; Additional file 3). In the DEG list, each probeset is accompanied by the three best matching potato TAs. For each TA, perfect alignment values, TA identification number and TA annotation are reported.

**Figure 1:**
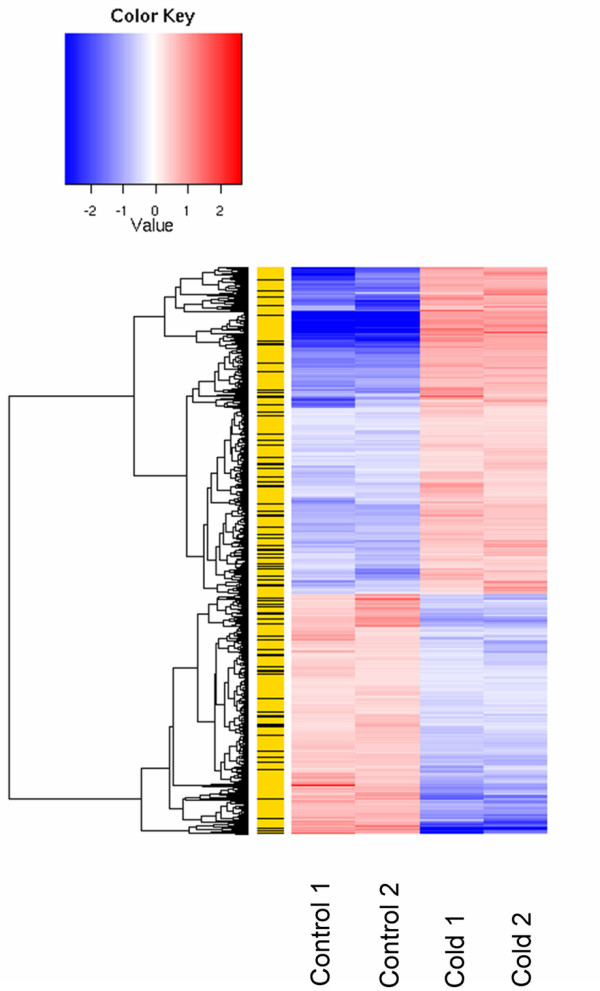
**Hierarchical clustering**. Clustering of all 1,854 differentially expressed probesets (control 17°C vs. 4 day cold incubation at 4°C, two biological replicates for each condition as indicated) and the intersection between these probesets and those over the 70% perfect alignment (%_PERF_ALIGN) threshold. The length of each branch of the dendrogram indicates (1 – Pearson) correlation coefficients as a measure of similarity. The column between the dendrogram and the heatmap represents the probesets either differentially expressed and selected with the 70% perfect alignment threshold (yellow) and the probesets differentially expressed but not selected by the perfect alignment threshold (black).

Out of the 1,854 DEG, 1,199 and 1,528 are conserved when filtered at thresholds > 90 and > 70, respectively. Figure 1 depicts the hierarchical clustering of all the 1,854 differentially expressed probesets and the intersection between these probesets and those above the 70%_PERF_ALIGN threshold. The probesets that are differentially expressed, but not selected by the 70%_PERF_ALIGN threshold, are flanked by black lines.

**Figure 2:**
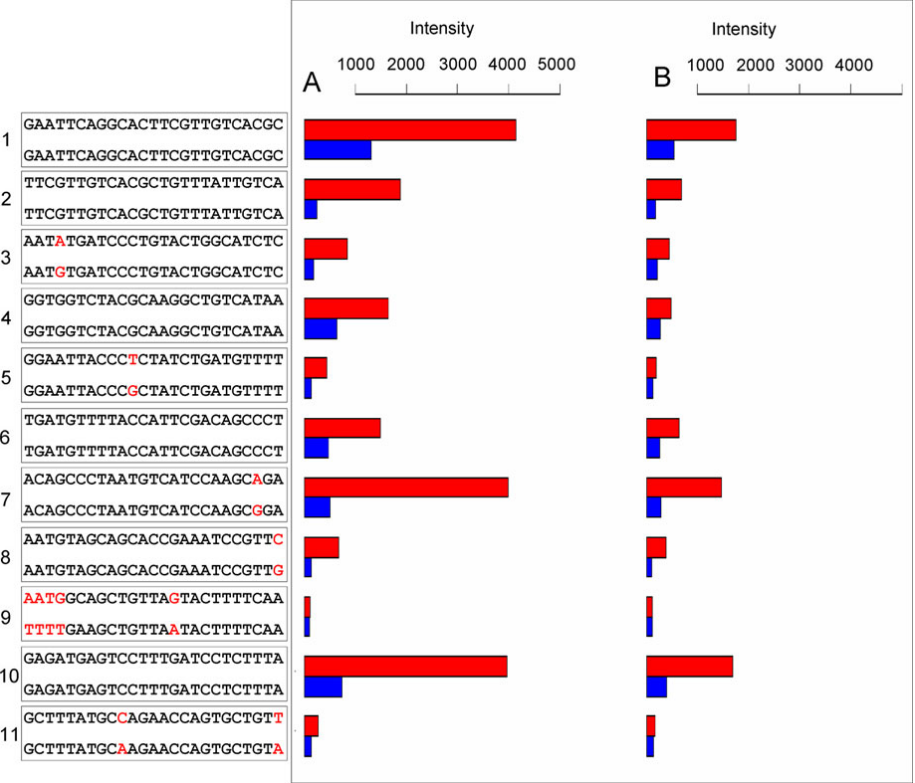
**Probe-level analysis of GeneChip signals as affected by sequence mismatches**. Signal intensity at a probe level in stress (A) vs control (B) GeneChip dataset as affected by sequence mismatches. SPS (Les.3522.1.S1_at; potato counterpart TA26174_4113; 2.96 fold induction) is shown as an example. The figure was generated as follows: based on the Global Match File, potato SPS (Transcript Assembly TA26174_4113) best aligns to the target sequence of probeset Les.3522.1.S1_at. Thus, the 11 probes (perfect match probes, PM) associated with this probeset (on the left, upper sequences) were aligned to TA26174_4113. The 25 nt-long potato subsequences that align to tomato probes are reported below the tomato probe sequences, numbered from 1 to 11. Mismatches are highlighted in red. Red and blue bars represent signal intensities associated with perfect match and mismatch probes, respectively. Similar intensities were obtained for the two biological replicates of control and cold conditions, but for the sake of clarity only one of the two GeneChip replicates is shown.

Figure 2 exemplifies how sequence mismatches affect GeneChip signal intensities at the level of single probes within probesets. SPS (Probeset Les.3522.1.S1_at; potato counterpart TA26174_4113; 2.96 fold induction) is listed in the 70% group (79% %_PERF_ALIGN value) but a further, high similarity region is present towards the 3' region (16% %_PERF_ALIGN) making a 95% total %_PERF_ALIGN score. As a general trend, probes with 100% match to potato sequences (1, 2, 4, 6 and 10) or just one peripheral mismatch towards 5' or 3' extremities (3, 7 and 8) showed the highest differences in cold vs. control datasets. The fact that probes 100% identical to potato sequences showed different absolute intensities is not surprising as various sequence-specific phenomena, including probe GC % composition, cross-hybridization and interference with mismatch (MM) probes are known to alter absolute hybridization efficiencies [3]. However, those differences are strongly mitigated when the intensity ratios between stress vs. control dataset rather than absolute values are compared. On the other hand, probes with central mismatches and/or more than one mismatch (5, 9 and 11) tended to exhibit severely altered signals and signal ratios. Only multiple or central mismatches affecting central probe regions dramatically altered signal detection (probes 9 and 11).

One of the many uses of the Global Match File is that keyword searches can immediately provide hits and related alignments pointing to probesets of interest. These are especially valuable as GeneChip tomato default annotations may be substantially less informative and/or updated. In these keyword searches, the > 90 and > 70 lists are particularly useful when a narrowing down of hits to best alignments is desired.

**Figure 3:**
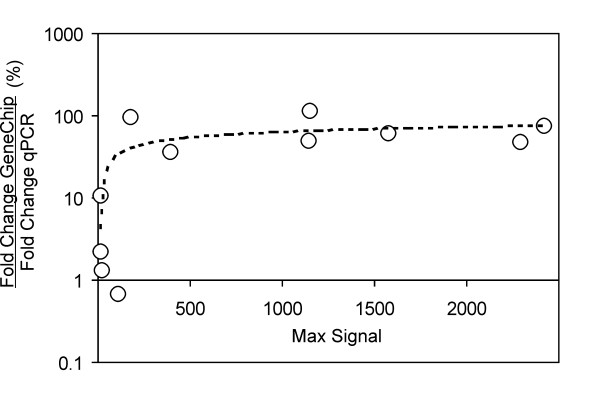
**Comparative evaluation of qPCR vs. GeneChip fold induction estimates**. The ratios of fold-induction values of DEG as measured by GeneChip dataset and qPCR are plotted as percentage values against "Max Signal" values, which represent the highest average GeneChip signals, both in the control and cold-stressed dataset.

Overall, the Global Match File proved efficient in identifying reliable potato counterparts. In fact, by filtering out matches below 70 %_PERF_ALIGN, no discrepancies could be found in up-regulation vs. down-regulation calls in the 11 genes further tested by qPCR (see also Fig 3). However, as discussed in the next section, in several cases GeneChip fold induction was less than that measured by qPCR.

### GeneChip vs. qPCR Data

Possibly as a consequence of mismatches between querying vs. target sequences, we noticed that GeneChip fold induction estimates compared better to qPCR data in cases of high "Max Signal" index. This parameter is equal to the maximum average chip signal (both in the control and cold-treatment chip datasets) and was chosen to roughly estimate overall transcript abundance.

In rare or less-abundant transcripts, fold-induction magnitudes appeared to be underestimated by GeneChip dataset. This is shown in Figure 3, where the GeneChip to qPCR fold induction ratio is plotted versus Max Signal parameters. This was taken into account when interpreting our GeneChip dataset and should possibly be considered in heterologous GeneChip approaches. Thus, it is likely that a disturbance factor inherent in the heterologous approach is a generalized lowering of the Max Signal parameter, which in turn may affect fold induction estimates in some extreme cases.

### Cold-associated genes

The first step in assessing the reliability of the heterologous GeneChip approach was to verify the up-regulation of known cold-inducible genes. It has been shown that cold can result in the induction of a broad spectrum of Heat-Shock genes [21,22]. In fact, several genes of this class were highly induced following a 4-day incubation at 4°C (Table 2). Table 3 details further known cold-responsive genes in our dataset, including dehydrin-class genes [23,24], Cu-Zn superoxide dismutase [25], alternative oxidase and plant uncoupling mitochondrial protein [26], omega-3 fatty acid desaturases [27], temperature-induced lipocalin, and various entries belonging to the class of Late Embryogenesis Abundant proteins (LEA) [28,29].

**Table 2:** Heat shock upregulated genes.

**Probeset ID**	**Potato best match and annotation**	**Avg. signal 17°C**	**Avg. signal 4°C**	**Fold change**	**Adj. P.Val.**
Les.269.1.S1_at	TA34173_411 Small heat shock protein, chloroplast precursor (Tomato)	94.31	4072.03	**42.94**	0.00129
LesAffx.10596.1.S1_at	BG888211 Chloroplast small heat shock protein class I (*Capsicum frutescens*)	64.18	2561.36	**39.78**	0.00129
Les.4004.1.S1_a_at	TA25739_4113 Hsp19.9 protein (*Lycopersicon peruvianum*)	149.19	5743.57	**38.50**	0.00140
Les.4819.1.S1_at	TA24545_4113 DnaK protein, putative (*Oryza sativa*)	44.76	1295.63	**29.84**	0.00254
Les.3677.1.S1_at	TA35283_4113 Small heat-shock protein (Tomato)	16.02	396.81	**25.04**	0.00182
Les.4150.1.S1_at	TA29480_4113 Mitochondrial small heat-shock protein (Tomato)	173.18	1515.29	**8.75**	0.00287
Les.3160.3.S1_at	TA24543_4113 DnaK protein, putative (*Oryza sativa *– japonica cultivar-group)	173.64	1294.13	**7.39**	0.00325
Les.3739.1.S1_at	TA42758_4113 Small heat-shock protein homolog protein (*Solanum tuberosum*)	4.30	20.16	**4.18**	0.04704

Signals are the averages of two biological replicates (avg. signal) for both control (17°C) and cold stress (4 days at 4°C) and fold changes represent the ratios of stress to control average signals. For a given probeset, potato best match refers to the best hit (highest perfect alignment score) between tomato target vs. potato TIGR TA entry as determined by the Global Match File. Adjusted P-value (Adj.P.Val) estimates false positives and refers to Benjamini-Hochberg's multiple test correction of the false discovery rate.

**Table 3:** Selected, miscellaneous cold-responsive genes.

**Probeset ID**	**Potato best match and annotation**	**Avg. signal 17°C**	**Avg. signal 4°C**	**Fold change**	**adj.P.Val**
Les.4223.1.S1_at	TA28833_4113 Alternative oxidase 1au (*Solanum lycopersicum*)	4.79	330.56	**69.21**	0.00189
Les.3967.1.S1_at	TA32068_4113 Omega-3 desaturase (*Solanum tuberosum*)	18.98	471.64	**24.84**	0.00129
LesAffx.23510.1.S1_at	TA42248_4113 PvLEA-18 (*Phaseolus vulgaris*)	12.66	140.28	**11.01**	0.00298
Les.5956.1.S1_s_at	TA23754_4113 Cold-stress inducible protein (CI7) (*Solanum tuberosum*)	130.33	1406.81	**10.88**	0.00276
Les.3273.1.S1_at	BM113620 Cold acclimation protein WCOR518 (*Triticum aestivum*)	49.42	448.51	**9.00**	0.00321
Les.5011.1.S1_at	TA35430_4113 Temperature-induced lipocalin (*Solanum tuberosum*)	120.12	895.12	**7.45**	0.00239
Les.3341.1.S1_at	TA24917_4113 Copper-zinc superoxide dismutase (*Solanum tuberosum*)	2079.22	5038.78	**2.41**	0.01200
Les.3691.1.S1_at	TA30474_4113 Mitochondrial uncoupling protein (*Solanum tuberosum*)	33.01	75.79	**2.30**	0.01080
Les.5957.1.S1_at	TA36769_4113 Cold acclimation protein WCOR413-like protein beta form (*Arabidopsis thaliana*)	375.55	711.09	**1.89**	0.02172

Signals are the averages of two biological replicates (avg. signal) for both control (17°C) and cold stress (4 days at 4°C) and fold changes represent the ratios of stress to control average signals. Further details are as described in Table 2.

In addition to the above genes, a widespread reaction to chilling stimuli in plants is the accumulation of sugars, as they can act like compatible solutes fulfilling an osmoprotective role [30-32]. Enhanced mRNA levels for carbohydrate-associated genes accompany sugar increases [30], as confirmed in our GeneChip dataset and further ascertained by qPCR data (as detailed in the next section). Other genes that are frequently reported to be cold-responsive are flavonoid-associated as summarized in Table 4[32,33]. However, sugars and in particular sucrose are a well-established trigger for this pathway [34-37] and thus cold may only indirectly mediate enhanced expression of flavonoid-associated genes via an increase in sugar levels. As a consequence of cold incubation, sucrose reaches levels that are known to influence transcript accumulation for the above genes in just a few days [36].

**Table 4:** Flavonoid- and anthocyanin-related genes.

**Probeset ID**	**Potato best match and annotation**	**Avg. signal 17°C**	**Avg. signal 4°C**	**Fold change**	**adj. P.Val**
Les.3649.1.S1_at	TA30979_4113 Chalcone synthase 2 (*Solanum tuberosum*)	6.96	3965.21	**576.52**	0.00128
Les.3650.1.S1_at	TA30759_4113 Chalcone synthase 1B (*Solanum tuberosum*)	15.17	2835.03	**221.07**	0.00391
LesAffx.68320.1.S1_at	TA41264_4113 Putative chalcone isomerase 4 (*Glycine max*)	6.97	1028.68	**146.95**	0.00128
Les.4271.1.S1_at	TA24485_4113 Phenylalanine ammonia-lyase (*Capsicum chinense*)	18.27	1845.22	**103.86**	0.00182
Les.2988.1.S1_at	TA25065_4113 Cinnamic acid 4-hydroxylase (*Capsicum annuum*)	84.47	1601.26	**18.92**	0.00182
Les.4412.1.A1_at	TA26173_4113 Leucoanthocyanidin dioxygenase 2, putative; 51024–52213 (*Arabidopsis thaliana*)	10.95	133.78	**12.19**	0.00209

Signals are the averages of two biological replicates (avg. signal) for both control (17°C) and cold stress (4 days at 4°C) and fold changes represent the ratios of stress to control average signals. Further details are as described in Table 2.

### Transcript profiling of carbohydrate-associated genes during early cold sweetening events

**Figure 4:**
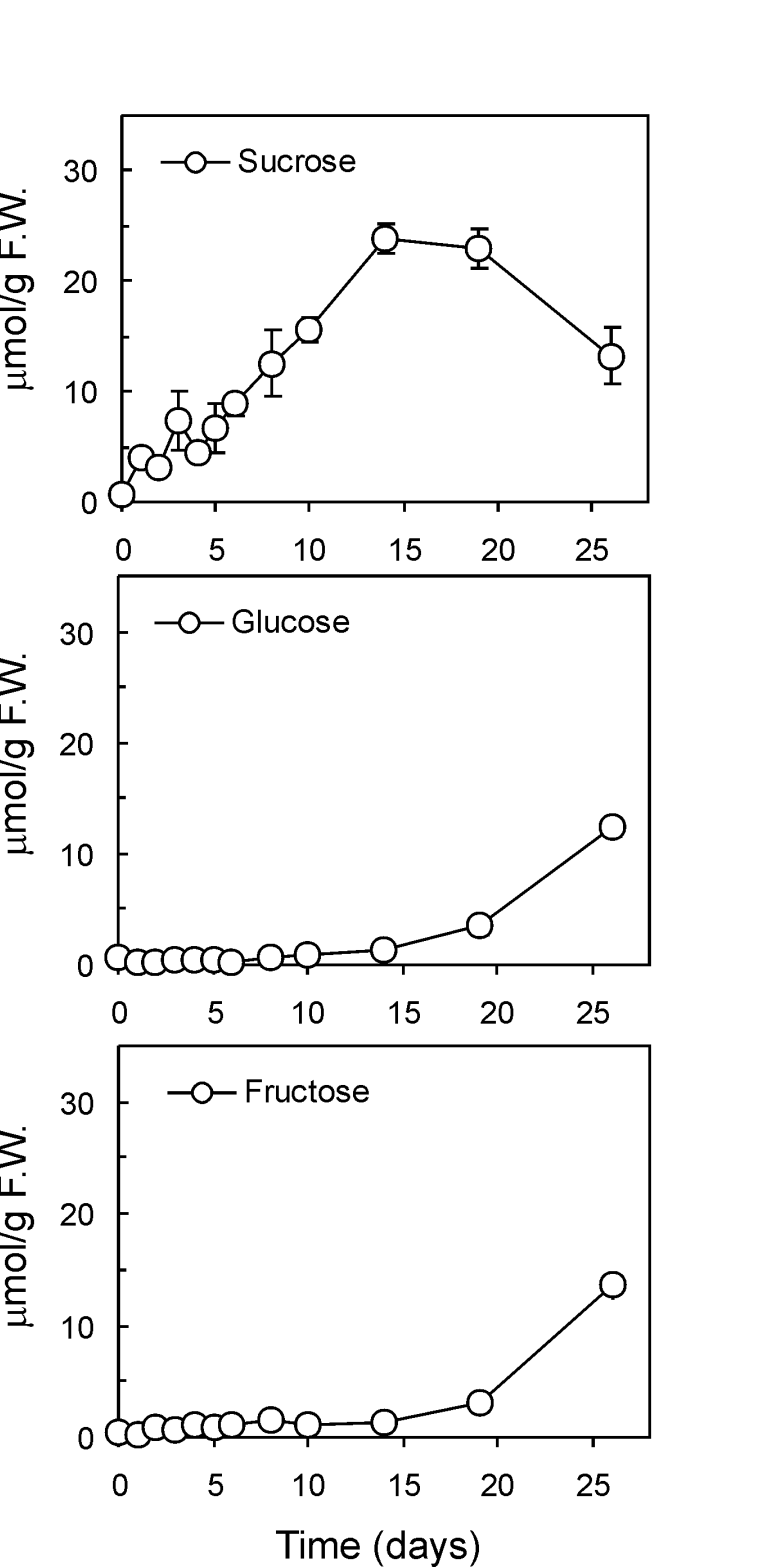
**Sucrose, glucose and fructose profiles in tubers incubated at 4°C**. Sucrose, glucose and fructose profiles in tubers incubated at 4°C. Sugar accumulation was monitored during the cold incubation (4°C; 26 days). For each time point, three independent tubers were analyzed and data are plotted as means ± SD.

We preliminarily investigated the pattern of sugar accumulation in a time course experiment spanning 26 days. Day 0 represents the 17°C control. Sucrose, glucose and fructose contents of three independent tubers for each time point were measured and their averaged values ± SD are plotted in Figure 4. In agreement with other studies detailing early cold sweetening events [38,39], sucrose began accumulating at the onset of cold incubation and reached a plateau within two weeks. Glucose and fructose accumulated slowly during the first few days and increased sharply as the sucrose levels stopped increasing. At the last sampling time (day 26) glucose and fructose levels equalled sucrose concentration on a molar basis.

**Figure 5:**
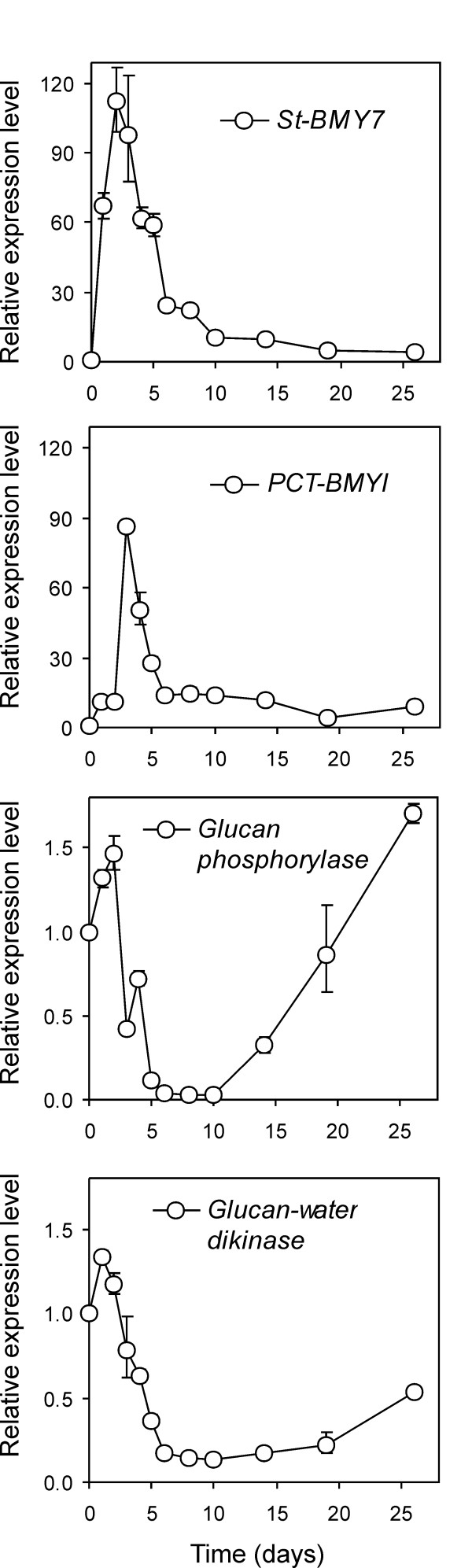
**Expression pattern of starch-associated genes as measured by qPCR**. Transcript accumulation of St-BMY7 (TA23155_4113, showing best homology to *Arabidopsis *TRX-regulated β-amylase BMY7); PCT-BMYI (TA33682_4113; potato chloroplast-targeted β-amylase PCT-BMYI); glucan phosphorylase (TA24089_4113) and glucan-water dikinase (TA25853_4113) upon tuber incubation at 4°C was monitored over 26 days. The Relative Expression Level (REL) is reported (REL of the control, 17°C tubers equals 1).

It is generally accepted that cold sweetening is fuelled by starch-derived hexoses, and various studies have been carried out to investigate the contribution of starch-degrading enzymes. Some studies have reported an involvement in CIS of Glucan phosphorylases, α and β-amylases, and α-glucosidases [14]. Glucan phosphorylase has long been argued as being responsible for cold-triggered starch degradation [[14] and references therein] and transgenic antisense approaches, including recent ones, have succeeded to some extent in lowering glucose accumulated over three months of cold storage [40]. However, several studies do not support the prominent role of phosphorolytic starch degradation in the early stages of cold storage, as opposed to other enzymes. In fact, at least one cold-triggered amylase activity was shown to be activated as highlighted by zymograms [38]. These amylolytic activities were identified as β-amylases based on substrate specificity [16] but still remained uncharacterized at a molecular level. Our GeneChip data indicated that a β-amylase (Les.2844.1.S1_at; best potato hit: TA23155_4113) underwent strong (61-fold) up-regulation. A qPCR Taqman assay based on the potato TA23155_4113 sequences is shown in Figure 5, indicating a strong up-regulation within the first two days, peaking at day 2 with a 112-fold induction and a subsequent decline to 4-fold induction at day 26. This potato TA showed maximum homology (82% identity and 92% homology based on TblastN output) to plastid-targeted *Arabidopsis *β-amylase *BMY7 *(*At3g23920*) known to undergo redox activation by thioredoxins and to be especially expressed in non-photosynthetic tissues in a stress-responsive manner [41].

However, TA23155_4113 was not full length and covered only about 70% of *Arabidopsis BMY7 *most 3' CDS. Nonetheless, all 6 Cys residues in the alignment were conserved, including the Cys-470, which has been suggested as forming an inhibitory disulfide with Cys-32 in *BMY7 *[41]. We thus analyzed Unigene cluster Stu.4927 whose 3' sequences perfectly matched potato TA23155_4113 and confirmed the presence in Stu.4927 of ESTs encompassing the start codon (e.g. CK274395) of all the remaining 2 Cys, including Cys-32. Furthermore, chloroP v1.1 [42] predicted a plastid transit peptide for the same 5' EST in Stu.4927 cluster. It seems therefore likely that TA23155_4113 and the more complete Stu.4927 unigene entries do represent the potato ortholog (which we propose to call *St-BMY7*) of *Arabidopsis BMY7*. Intriguingly, transcriptional up-regulation of *St-BMY7 *closely mirrored the β-amylase activity profiles shown by Hill et al. [38] and Nielsen et al. [16].

We next investigated the possible cold-dependent regulation of additional β-amylase mRNAs. Probeset LesAffx.53231.1.S1_at best matches *PCT-BMYI *(TA33682_4113), i.e. potato tuber chloroplast-targeted β-amylase (*Arabidopsis *homologue: *BMY8*; *At4g17090*; [43-45]), but showed only negligible induction (1.1 fold). Nonetheless, the very low signal intensity (Max Signal equal to 14) coupled with moderate identity despite the otherwise full alignment (95% identity within alignment, as assessed by the Global Match File) suggested that further probe level analysis was necessary to rule out significant up-regulation of this further β-amylase. Indeed, probe match analysis revealed that only probes 8 and 9 matched perfectly, and subsequent visualization at a probe level highlighted that some higher up-regulation was indeed occurring (data not shown). We thus tested *PCT-BMYI *transcript levels by qPCR confirming significant early up-regulation, peaking on day 3 but still sustained at day 26 (9-fold induction; Figure 5).

Overall, our data indicate that at least two cold-triggered β-amylase activities can be identified at the sequence level with TA23155_4113 (which we propose to call *St-BMY7*) and *PCT-BMYI *i.e. potato tuber chloroplast-targeted β-amylase [43]. As in previous studies only one or at best two β-amylases were visible in zymograms [16,38], we believe that most of the early, cold-triggered β-amylase activity has now been accounted for. The finding that an *Arabidopsis BMY7 *homologue was induced by cold may have important implications in terms of a redox regulation of early amylolytic events and the overall contribution that such redox signalling can exert over the entire sweetening process. Intriguingly, the activation of the oxidative pentose phosphate pathway and thus the fuelling of NADPH for redox-controlled processes was supported by transcript accumulation of several dedicated genes. Some of these entries are listed in Table 5. In particular, transcripts for a plastid-targeted Glc 6-P dehydrogenase (Les.824.1.S1_at; TA30812_4113) are 2.8-fold induced. This dehydrogenase belongs to the P2-type group showing reduced NADPH feedback inhibition and redox modulation, and thus seems most appropriate for determining a NADPH upsurge to cope with redox processes in a heterotrophic tissue [46]. Similarly, 6-phosphogluconate dehydrogenase (Les.2101.1.A1_a_at; TA27126_4113) transcript was 3.4-fold induced. Although the tomato GeneChip may not represent some gene family members and some homologies may be too poor to disclose full up-regulation, several additional transcripts encoding genes involved in thiol signalling are indeed upregulated, such as several Trx homologs [including an *m*-type Trx, recently observed in amyloplasts isolated from wheat starchy endosperm by Balmer et al., [47] and ferredoxin-NADP+ reductase and ferredoxin-Trx-reductase [Table 5]]. A non-photosynthetic ferredoxin type III [48] is induced as well (Les.1990.2.A1_at; 2.32-fold). Amyloplasts contain a complete battery of enzymes for redox thiol signalling, including ferredoxin-NADP reductase, ferredoxin, ferredoxin-Trx reductase (FTR), and Trx (*m*-type). It thus appears that amyloplasts can translate a sugar signal to a redox signal for further, thiol-mediated redox post-translational regulation [46,49,50]. However, such activation has not been reported for *PCT-BMYI*. One explanation may be that the two transcriptionally activated β-amylases, *St-BMY7 *and *PCT-BMYI*, exhibit complementary features. The first enzyme is regulated by redox cues, while *PCT-BMYI *is capable of hydrolysing, in addition to soluble starch, intact potato starch granules [43]. Furthermore, at the very end of our sampling time, *PCT-BMYI *transcripts were still tenfold higher than the control, suggesting persisting transcriptional activation even at later stages of cold incubation.

**Table 5:** Redox and thiol-signalling upregulated genes

**Probeset ID**	**Potato best match and annotation**	**Avg. signal 17°C**	**Avg. signal 4°C**	**Fold change**	**Adjusted P. Value**
**Les.1250.2.S1_at**	TA31380_4113 Thioredoxin-like protein; (*Arabidopsis thaliana*)	18.55	76.16	**4.11**	0.00466
**Les.3533.1.S1_at**	TA28486_4113 Non-photosynthetic ferredoxin (*Ipomoea nil*)	358.72	1338.27	**3.74**	0.00544
**Les.2101.1.A1_a_at**	TA27126_4113 6-phosphogluconate dehydrogenase, putative (*Arabidopsis thaliana*)	85.67	291.78	**3.40**	0.00706
**Les.3221.1.S1_at**	TA29395_4113 Thioredoxin (*Solanum berthaultii*)	135.03	409.47	**3.04**	0.00751
**Les.824.1.S1_at**	TA30812_4113 Glucose-6-phosphate 1-dehydrogenase precursor (*Solanum tuberosum*)	64.19	169.95	**2.80**	0.04315
**Les.1990.2.A1_at**	TA32815_4113 Ferredoxin-3, chloroplast precursor (*Zea mays*)	8.92	21.09	**2.32**	0.03972
**Les.4466.1.S1_at**	TA35589_4113 Subunit A of ferredoxin-thioredoxin-reductase precursor (*Solanum tuberosum*)	14.64	30.82	**2.09**	0.04161
**Les.194.1.S1_at**	BQ113042 Putative thioredoxin (*Solanum lycopersicum*)	6.53	12.95	**1.98**	0.01720
**Les.2479.1.S1_at**	TA33226_4113 Thioredoxin M-type, chloroplast precursor (*Brassica napus*)	50.34	98.15	**1.97**	0.03015
**Les.1129.1.S1_at**	TA28250_4113 Ferredoxin-NADP reductase, root isozyme, chloroplast precursor (*Pisum sativum*)	63.73	121.88	**1.93**	0.02755

Signals are the average of two biological replicates (avg. signal) for both control (17°C) and cold stress (4 days at 4°C) and fold changes represent the ratios of stress to control average signals. Further details are as described in Table 2.

We then investigated other amylolytic enzymes. Glucan phosphorylase has been widely mentioned in the cold-sweetening process as being involved in starch breakdown [14]. Two probesets, namely Les.2820.1.S1_at and Les.2820.2.S1_at with best hit TA24089_4113, coding for potato glucan phosphorylase (α-1,4 glucan phosphorylase, L-1 isozyme, chloroplast precursor) showed down-regulation at day 4 of cold-incubation (2.94- and 3.22-fold, respectively, with the first probeset included in the DEG list). qPCR confirmed moderate transcript down-regulation, which after a negligible induction in the first two days started decreasing with an intervening minor peak on day 4 down to a more than 30-fold decrease on days 8 and 10 (Figure 5). The transcript level then rose steadily to reach a 1.8-fold induction, suggesting that transcriptional regulation of glucan phosphorylase, if present, may only be relevant at later stages. Glucan-water-dikinase (GWD) is another key enzyme controlling the phosphorylation degree of starch and its susceptibility to degradation, possibly as a consequence of relaxed steric hindrance and increased accessibility of amylolytic enzymes [51]. *Arabidopsis *defective for the GWD homolog gene (SEX1; *STARCH EXCESS 1*) exhibited a starch over-accumulating phenotype and reduced freezing tolerance and *SEX1 *transcripts were also cold induced [52]. Furthermore, some transgenic antisense approaches with GWD were successful in reducing level of sugars in tubers following a two-month-long incubation at 4°C [53]. Probesets Les.3195.1.S1_at and Les.3195.2.S1_at (best hit for both TA25853_4113, potato GWD, chloroplast precursor) were both included in the DEG list (1.96 and 2.12 -fold down-regulation, respectively). Figure 5 shows that the time course for GWD transcript accumulation was similar to glucan phosphorylase with a net decrease approaching one-tenth of the control levels after one week and a subsequent rise to half that of the control levels at the last sampled time. Overall, these two genes showed moderate transcriptional regulation by cold at least within the experimental time frame of 26 days. However, both enzymes are known to be redox-regulated by thioredoxins [47] and thus post-transcriptional events may alter this scenario. On the other hand, activity measurements on zymograms have failed to detect glucan phosphorylase activity modulation at least in the first 48 days [38].

We also analysed invertases, as they convert sucrose to the reducing sugars glucose and fructose. Invertases have been extensively investigated in the context of potato cold sweetening, and several studies have concluded that these enzymes mainly control the hexose/sucrose ratio, ruling out a more general influence on overall sugar accumulation [14]. Poor correlation was in fact found among total invertase activity (both acid and neutral) and overall sugar accumulation [19]. The scenario is however rather intricate as an invertase inhibitor may alter the overall activities [54]. Using northern blots Zrenner et al. [19] monitored the accumulation of transcripts coding for a soluble acid invertase (*Pain-1*), and detected a strong accumulation after tuber storage at 4°C, rising steadily after 2 days up to two weeks, until a decline was observed after 6 weeks. A transgenic antisense approach with the same acid invertase solely resulted in a lowering of hexoses, which was however paralleled by an increase in sucrose levels after tuber cold-incubation for 20 weeks. A transgenic approach involving ectopic expression of a tobacco invertase inhibitor circumvented the problem of possible invertase isoforms and resulted in a fair reduction (up to 75%) of hexoses [55]. Nonetheless, as noted elsewhere, cold-induced reducing sugar accumulation was still noticeably high to comply with processing industry requirements [14].

**Figure 6:**
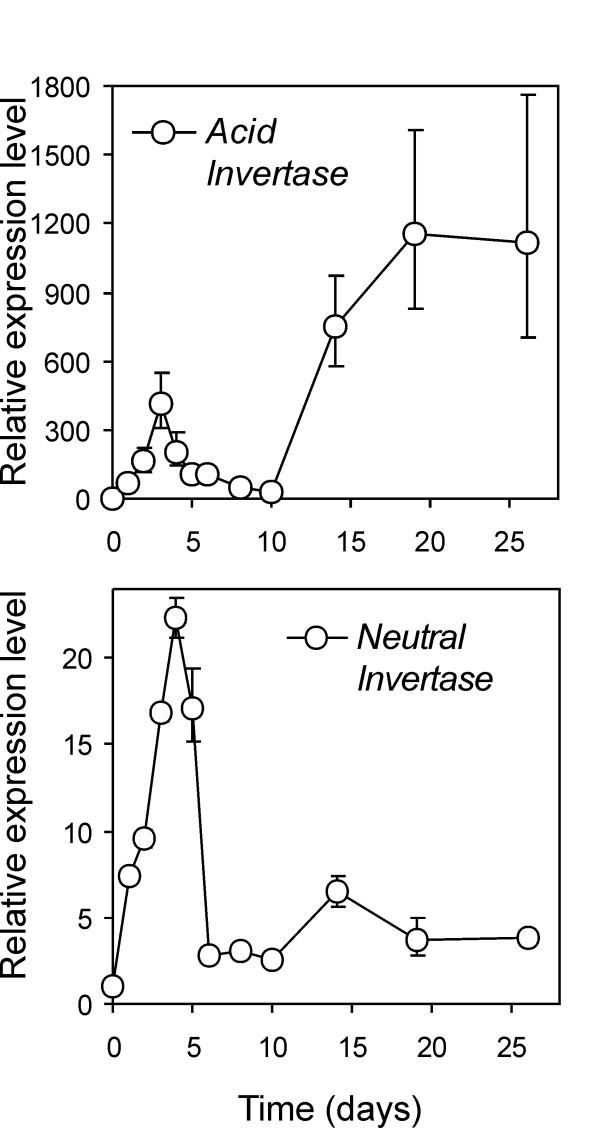
**Expression pattern of invertases as measured by qPCR**. Transcript accumulation of vacuolar acid invertase Pain-1 (TA26908_4113) and neutral invertase (TA42474_4113, "putative neutral/alkaline invertase, Cassava") upon tuber incubation at 4°C was monitored over 26 days. The Relative Expression Level (REL) is reported (REL of the control, 17°C tubers equals 1).

The existence of other cold-induced invertases has been widely proposed. Several cold-induced invertases with distinct properties such as pH-dependence, *K*_*m *_and heat-stability could be separated by column chromatography [56]. Our GeneChip data revealed that only two probesets referring to invertases were differentially regulated. The first one, Les.2702.1.S1_a_at, despite an intervening stop codon in the tomato target region influencing the overall alignment and a low Max Signal (Max Signal value equal to 20), was included in the DEG list and was 2.7-fold induced. The probeset best refers to potato TA26908_4113, annotated as potato acid invertase, which in turn refers to invertase *Pain-1 *[19]. qPCR with *Pain-1 *specific Taqman assay revealed a biphasic induction pattern, with 400-fold induction peaking at day 3 and, following a slight decline to a 30-fold induction, again increased to reach an apparently stable plateau (1100-fold) until the final sampling time (Figure 6). This pattern may not really be distinct from the *Pain-1 *transcript pattern described above, as the early induction peak may not have been observed in the previous analysis due to fewer samplings in the 4- to 14-day time window [19].

A second probeset (LesAffx.37983.1.S1_at, corresponding to potato TA42474_4113 annotated as "putative neutral/alkaline invertase Cassava") showed a 2.37 fold induction. Although not listed in our DEG list, the very low Max Signal level (Max Signal value equal to 15) suggested that some underestimation might have occurred. Indeed, probe level analysis confirmed induction as assessed by signals associated to perfectly matching probes (not shown). As cold-triggered transcript up-regulation for an invertase has only previously been reported for the acid invertase *Pain-1*, we monitored neutral invertase transcripts by qPCR. As shown in Figure 6, we confirmed up-regulation (24-fold induction at day 4) followed by a decline to levels slightly above the controls (3- to 4-fold induction) in the remaining sampling times. The partial success previously obtained with transgenic *Pain-1 *acid invertase antisense approaches over total sugar accumulation may be due to the expression of this additional neutral invertase.

**Figure 7:**
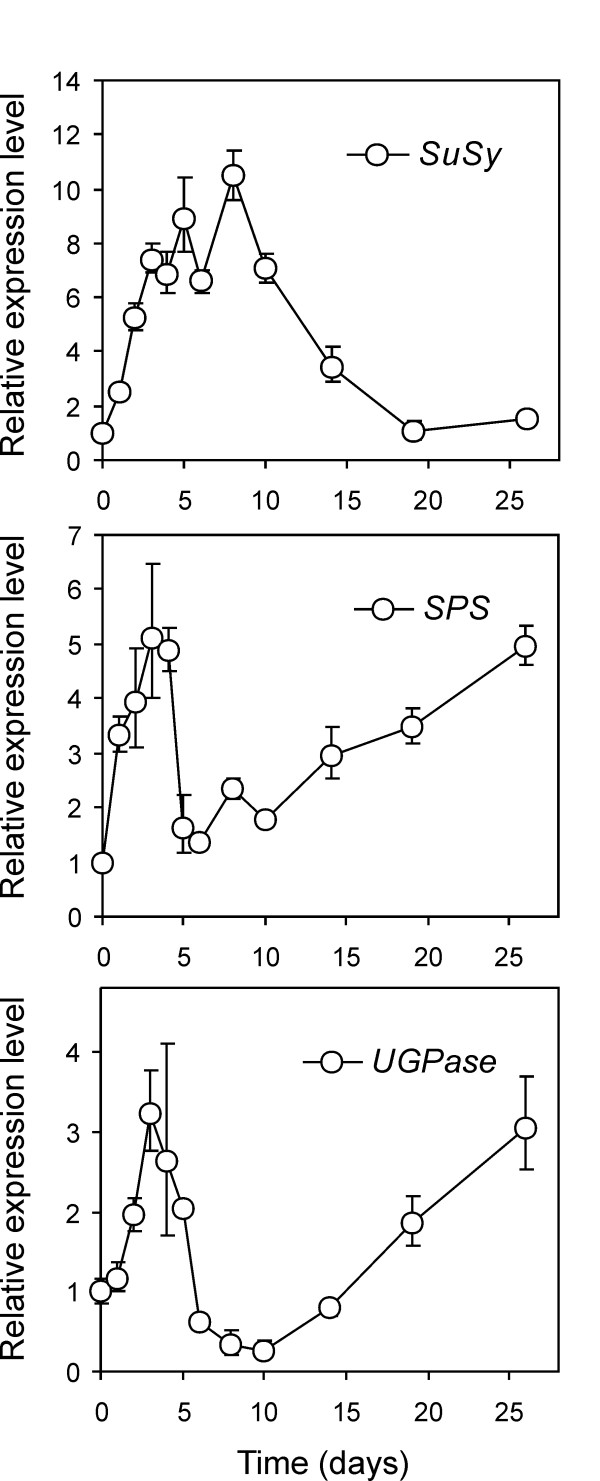
**Expression pattern of sucrose-associated enzymes as measured by qPCR**. Transcript accumulation of sucrose synthase (SuSy; TA26908_4113), sucrose phosphate synthase (SPS; TA26174_4113) and UDP-glucose pyrophosphorylase (UGPase; TA24502_4113) upon tuber incubation at 4°C was monitored over 26 days. The Relative Expression Level (REL) is reported (REL of the control, 17°C tubers equals 1).

UDP-Glucose-pyrophosphorylase (UGPase) catalyses the formation of the high energy compound UDP-Glc which, with Fru-6-P, is the substrate for sucrose synthesis and therefore is of paramount importance for sweetening. Despite the presence of various isoforms which have been involved in conferring differential susceptibility to CIS [14,57], the enzyme is poorly regulated, and antisense approaches have revealed that as little as 4–5% of UGPase expression was sufficient to carry out normal functions, as the tuber did not show relevant changes in carbohydrate-associated parameters [14]. In any case, steady-state levels of UGPase mRNA were found to be very high in sink tubers, and dropped dramatically following harvest and prolonged storage (8 months) in the dark at room temperature. Cold storage of these tubers led to a significant increase in steady-state mRNA levels compared to tubers stored for 8-months at room temperature at weeks 1 and 2, with a slight decrease at week 3 [18]. Tomato GeneChip Probeset Les.3208.1.S1_at refers to UGPase (potato TA24502_4113), and showed a minor (1.3-fold) up-regulation following the 4-day cold treatment. A moderate, but clear, induction was observed in qPCR peaking at day 3, followed by a decline to one-tenth of the control transcript and a subsequent, steady rise which continued to the final time point (Figure 7). Again, this profile is similar to previous northern experiments [18] when taking into account the less frequent sampling times used by these authors.

Sucrose Phosphate Synthase (SPS) has been widely studied in the context of sweetening. SPS is a highly regulated enzyme, undergoing modulation by allosteric effectors (Glc6P and Pi) and phosphorylation. Furthermore, SPS seems to alter its kinetic properties rather than its amounts of protein as a consequence of cold incubation and, as it is thought to be expressed in excess over cell demand, its activity is substrate-limited [58]. Nonetheless, it has been reported that SPS may undergo transcriptional activation since after 1, 2 or 3 weeks of 4°C storage, a constant increase in steady-state mRNA levels was observed [59]. Probeset Les.3522.1.S1_at is included in the DEG list (2.96-fold induction) and best matches potato TA26174_4113 annotated as *Solanum tuberosum *SPS. qPCR analysis showed an early induction peak (day 3; 3.5-fold) followed by a decline and again a stepping up which was sustained until day 26 (Figure 7). This data is in good agreement with previous northern experiments and similarly to those cases the first sampling time (1 week) was too late to detect the early burst of transcript accumulation [58].

GeneChip scrutiny indicated that probeset Les.157.1.S1_at, with the highest similarity to potato TA24067_4113 annotated as potato sucrose synthase 2, was 6.75-fold induced. As sucrose synthases are thought to mainly function in the sucrolytic direction, with a key role in determining tuber sink strength [60], comparatively fewer studies on sucrose synthase (SuSy) have been conducted in the context of potato tuber cold sweetening. As shown in Figure 7, SuSy transcript shows a relatively slower induction, possibly resulting from the sum of two early peaks. Maximum fold induction (10-fold) was at day 8 and was followed by a slow decline to values closely resembling control at the last time point. Since some SuSy gene family members contain sucrose-responsive elements [61], transcriptional activation may be triggered by sucrose accumulation, which is sustained within the first two weeks. The possible sucrose-dependent gene expression occurring in tubers after four days of cold storage is further supported by the induction of patatin, (Les.4742.1.S1_at; TA23313_4113; 6.46-fold), the abundant vacuolar storage protein bearing a well-characterized sucrose-responsive (SURE) element in its promoter [61]. SURE elements respond to, among others, WRKY transcription factors, which are in turn sugar-inducible [62], and at least two WRKY factors are upregulated based on our GeneChip data upon cold storage. These are "WRKY transcription factor 41 (*Oryza sativa*)"; (LesAffx.4793.1.S1_at; TA44358_4113; 3.16-fold) and "Transcription factor CaWRKY1 (*Capsicum annuum*)"; (LesAffx.21820.1.S1_at; TA33436_4113; 2.46-fold). This indicates that cold-associated sugar accumulation in potato tubers may in turn trigger the activation of a variety of sugar-responsive genes (e.g. the previously mentioned flavonoid- and anthocyanin-associated genes) thus branching outside carbohydrate-pertinent processes.

### Ethylene and fruit-ripening associated genes

The potato counterparts of numerous tomato ethylene biosynthesis, ethylene responsive and, more in general, fruit ripening-modulated genes were upregulated as a consequence of cold incubation for 4 days at 4°C. Table 6 lists the tomato probesets, fold induction, associated potato TA/singleton and annotation (best hit according to Global Match File) and reference material. Where applicable, the probeset entry refers to the tentative consensus ID and relative annotation of a recently produced list of ripening up-regulated transcripts (tomato ripening induced genes) available in the Tomato Expression Database (TED) [63-65]. The genes in Table 6 are listed according to their fold induction and only consist of a subset of representative ripening-upregulated transcripts selected to cover various biological processes. With respect to genes for ethylene biosynthesis, a probeset referring to potato *ACC *oxidase *ACO1 *gene (Les.2560.1.S1_at; potato best match TA25537_4113, annotated as potato ACC oxidase ACO1) was strongly upregulated (13-fold). A gene family member of ACC synthase (Les.3769.1.S1_at; best match TA42274_4113 annotated as potato ACC synthase) was only moderately induced (1.4-fold) and is not listed in the DEG. This may be due to known post-transcriptional regulation of ACS [66,67] or to the fact that ACO and ACS expression profiles frequently differ in timing [68-71]. Similarly, ACO but not ACS transcripts were found to strongly accumulate in mature green vs. breaker tomato fruit transition stages in the ripening-upregulated list [TED database [64]].

**Table 6:** genes transcriptionally up-regulated by both ripening and cold.

**Probeset ID**	**Avg. signal 17°C**	**Avg. signal 4°C**	**Fold change**	**Adjusted P. Value**	**Best potato match**	**Annotation**
Les.1936.1.S1_at	11.58	1990.21	**171.51**	0.00129	TA27416_4113	Early light inducible protein (*Solanum lycopersicum*) [64]; TC116636
Les.4223.1.S1_at	4.79	330.56	**69.20**	0.00189	TA28833_4113	alternative oxidase 1a (*Solanum lycopersicum*) [64]; TC116692
Les.4829.1.S1_at	14.26	586.14	**40.29**	0.00189	CV498197	2-oxoglutarate-dependent dioxygenase (*Solanum lycopersicum*) [64]; TC115851
Les.3338.1.S1_at	7.75	222.86	**29.98**	0.00300	TA24335_4113	S-adenosyl-L-methionine synthetase 1 (*Daucus carota*) [105]
Les.220.1.S1_at	8.10	226.46	**28.2**	0.00182	TA25585_4113	AER (*Nicotiana tabacum*) [106]
LesAffx.49935.1.S1_at	20.28	510.32	**26.62**	0.00384	TA28438_4113	Pyruvate decarboxylase (*Solanum tuberosum*) [64]; TC1163
Les.3551.1.S1_at	17.51	390.08	**22.9**	0.00255	CV475083	Ethylene-responsive transcriptional coactivator (ER24)(*Solanum lycopersicum*) [76]
Les.2988.1.S1_at	84.47	1601.26	**18.92**	0.00182	TA25065_4113	Cinnamic acid 4-hydroxylase (*Capsicum annuum*) [64]; TC124119
LesAffx.44987.1.S1_at	13.27	222.49	**16.76**	0.00175	TA38177_4113	Solanesyl diphosphate synthase (Hevea brasiliensis (Para rubber tree) geranyl diphosphate synthase (*Arabidopsis thaliana*) [64]; TC116882
Les.3651.1.S1_at	108.84	1738.28	**16.03**	0.00224	TA24332_4113	S-adenosyl-L-methionine synthetase (*Nicotiana tabacum*) [107]
Les.2560.1.S1_at	7.42	103.61	**13.90**	0.00226	TA25537_4113	Ethylene-forming enzyme (*Solanum lycopersicum*) [64]; TC123934
Les.4412.1.A1_at	10.95	133.78	**12.10**	0.00209	TA26173_4113	Leucoanthocyanidin dioxygenase 2, putative (*Arabidopsis thaliana*) [108]
Les.5956.1.S1_s_at	130.33	1406.81	**10.88**	0.00276	TA23754_4113	Cold-stress inducible protein (CI7) (*Solanum tuberosum*) [64]; TC116013
Les.4150.1.S1_at	173.18	1515.29	**8.74**	0.00287	TA29480_4113	Mitochondrial small heat-shock protein (*Solanum lycopersicum*) [64]; TC124001
Les.5826.1.S1_at	136.17	1105.79	**8.12**	0.00226	TA28749_4113	Cathepsin B-like cysteine proteinase (*Solanum tuberosum*) [64]; TC124060
LesAffx.1959.1.S1_at	28.53	224.93	**7.88**	0.00224	TA25003_4113	Glutathione S-transferase. class-phi (*Solanum commersonii*) [64]; TC116034
LesAffx.47187.1.S1_at	41.05	293.51	**7.14**	0.00236	TA36088_4113	Universal stress protein/early nodulin ENOD18-like [64]; TC123970
Les.3171.3.S1_a_at	18.56	120.71	**6.50**	0.00249	TA30735_4113	Phytoene synthase. chloroplast precursor (*Capsicum annuum*) [64]; TC115970
Les.3813.1.S2_at	142.73	855.96	**6.38**	0.01985	TA23078_4113	S-adenosylmethionine decarboxylase proenzyme [64]; TC123879
Les.3085.1.S1_at	28.13	178.22	**6.28**	0.00391	BG888309	Flavonol synthase/flavanone 3-hydroxylase (*Solanum tuberosum*) [64]; TC116718
Les.3285.1.S1_at	311.71	1908.05	**6.13**	0.00384	TA27830_4113	Acyl carrier protein (*Capsicum chinense*) [64]; TC124728
LesAffx.34336.1.S1_at	65.09	382.59	**6.12**	0.00895	TA46602_4113	70 kD heat-shock protein (*Arabidopsis thaliana*) [64]; TC126413
Les.4573.1.S1_at	28.35	150.86	**5.50**	0.00889	TA28898_4113	Guanylate kinase (*Nicotiana tabacum*) [64]; TC115887
LesAffx.69795.1.S1_at	27.80	148.00	**5.32**	0.00458	TA37100_4113	Putative methionine sulfoxide reductase B (*Oryza sativa*) [109]
Les.274.1.S1_at	8.38	29.61	**3.53**	0.01158	BQ046569	Ripening regulated protein DDTFR10/A (*Solanum lycopersicum*) [64]; TC116368
Les.824.1.S1_at	64.19	169.95	**2.79**	0.04315	TA30812_4113	Glucose-6-phosphate 1-dehydrogenase precursor (*Solanum tuberosum*) [64]; TC116406
Les.2702.1.S1_a_at	7.53	20.97	**2.66**	0.03637	TA26908_4113	Acid invertase (*Solanum tuberosum*) [64]; TC123895
Les.205.1.S1_at	174.85	461.58	**2.64**	0.00847	TA24762_4113	14-3-3 protein (*Solanum tuberosum*) [64]; TC116260
Les.2992.1.S1_a_at	87.62	225.46	**2.58**	0.01367	BQ504492	Callus-expressing factor (*Nicotiana tabacum*) JERF3; [99]
Les.3933.1.S1_at	1933.65	4519.78	**2.33**	0.00978	TA24146_4113	Adenosylhomocysteinase (*Solanum lycopersicum*) [64]; TC116044
LesAffx.10807.1.S1_at	18.17	35.73	**1.96**	0.01649	TA28665_4113	Heat-shock cognate 70 kDa protein (*Petunia hybrida*) [64]; TC126297
LesAffx.32723.1.S1_at	40.30	68.27	**1.69**	0.03380	TA26759_4113	BZIP transcription factor ATB2 (*Glycine max*) [64]; TC124112
Les.283.1.S1_at	138.53	228.92	**1.65**	0.03786	CV477421	Induced stolon tip protein (*Capsicum annuum*) [64]; TC124196
Les.303.1.S1_at	5852.89	9264.98	**1.57**	0.04951	TA24257_4113	Enolase (*Solanum lycopersicum*) [64]; TC123931

Selected entries from a set of genes, which are transcriptionally up-regulated by both ripening (various databases as indicated) and cold (our GeneChip dataset). TED database [64] refers to a recent list of tomato ripening-up-regulated genes (transition from mature green to breaker stage) and the ID of the corresponding tomato Tentative Consensus (TC) entry is reported. Fold changes represent the ratios of stress to control average signals (two biological replicates for both control and stress conditions). Further details are as described in Table 2.

**Figure 8:**
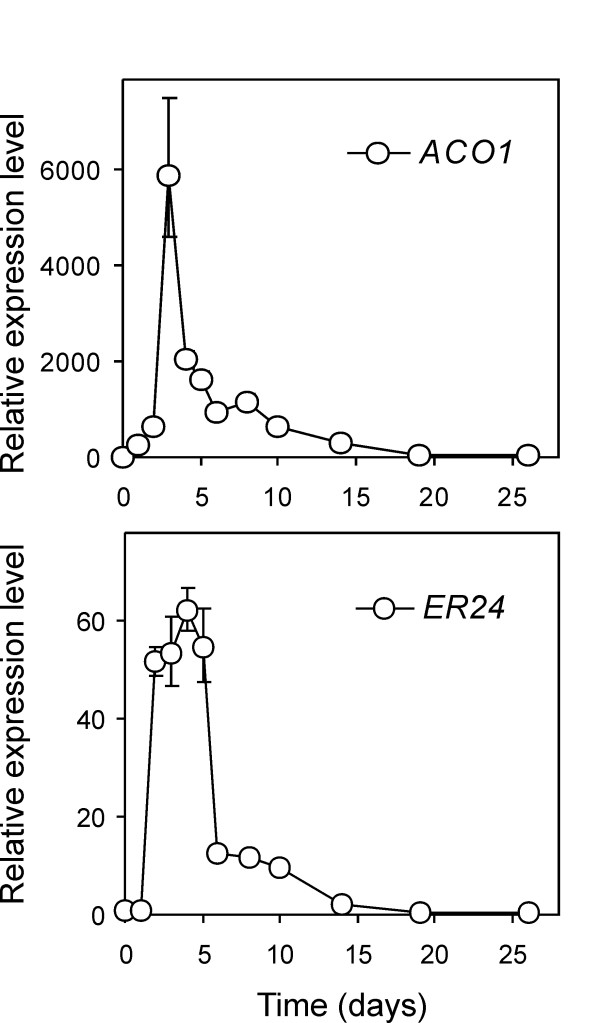
**Expression pattern of ethylene-associated enzymes as measured by qPCR**. Transcript accumulation of ACO1 (TA25537_4113) and ER24 (CV475083) upon tuber incubation at 4°C was monitored over 26 days. The Relative Expression Level (REL) is reported (REL of control, 17°C tubers equals 1).

To confirm the GeneChip data, we monitored potato *ACO1 *transcripts by qPCR. A dramatic transcript accumulation was detected which peaked at day 3 with a 5,800-fold induction. Transcript levels were subsequently lowered with an intervening shoulder at day 8 which preceded a further decrease. However, at the last sampling time point, ACO1 transcripts were still 43 times higher than the baseline (Figure 8).

Further early events known to occur following exposure to ethylene include the accumulation of transcripts for some ethylene receptors [72,73]. Indeed, transcripts for two ethylene receptors (Les.36.1.S1_at; CN214325 and Les.3490.1.S1_at; TA42479_4113) accumulate 3.30- and 4.27-fold, respectively, as a consequence of cold incubation in tubers. Several additional components of the downstream ethylene signalling machinery are known to undergo prevalent post-transcriptional regulation. These include the EIN3 transcription factor, which lies downstream of the ethylene receptors, and the negative regulator CTR1 and activates a wide range of ethylene-responsive transcription factors belonging to the ERF/AP2 group [74]. EIN3 protein steady state levels are tightly regulated by an ubiquitin/proteasome pathway mediated by the F-box proteins EBF 1 and 2 (EIN3-binding F-box proteins) [75]. While EBF2 is not represented in the tomato GeneChip, the potato homologue of tomato EBF1 (Les.411.2.A1_at; TA30136_4113) is 2.21-fold induced, mirroring what has been reported for the *Arabidopsis *EBF homologs upon ethylene stimulation [75].

In order to assess up-regulation of ethylene-induced genes in downstream steps, we tested the expression pattern of ER24 [76] by qPCR, an ethylene-responsive factor bearing similarities to transcriptional co-activators (Les.3551.1.S1_at; CV475083; GeneChip induction of 22.9-fold). ER24 transcripts increased at day 2 of cold incubation reaching a 60-fold induction until day 6 when they started to decline to tenfold with respect to the control. At day 26, ER24 transcripts were reduced to half that of the controls (Figure 8).

Various further ethylene-responsive transcription factors belonging to the ERF/AP2 were induced on the basis of our data set. To name just a few, JERF3 (Jasmonate and ethylene-responsive factor 3; Les.2992.1.S1_a_at; BQ504492; 2.58-fold), CaERFLP1, and Pti6, as further detailed below.

In conclusion, taking into account that a variety of ethylene-responsive genes whose induction kinetics are different from our GeneChip sampling time (4 days at 4°C) would have escaped detection and that the tomato GeneChip contains only 10,000 elements (and further entries are lost due to poor potato-tomato homology), various ethylene and ripening-induced genes in tomato and other climateric and non-climateric plants appear to be induced by cold in potato tubers.

No role, to our knowledge, has to date been attributed to endogenous ethylene in triggering/signalling potato tuber cold sweetening. However, in addition to our data, some circumstantial evidence supports this view. In fact, potato *ACO1 *and *ACO2 *have been shown to respond to various abiotic stresses including cold [77]. The production of "chilling ethylene" appears to be common in Solanaceous [[78] and references therein] and microarray studies reveal enhanced expression of numerous ethylene-responsive factors upon chilling treatment in pepper [79]. Similarly, exogenous ethylene administration to potato tubers closely mimics the effects of cold incubation. In fact, it causes the enhancement of CN-insensitive respiration, enhanced glycolysis and, where tested, the accumulation of sucrose and reducing sugars [[80-84], and references therein]. Studies on the exogenous ethylene anti-sprouting potential on potato tubers have determined that ethylene amounts need to be carefully modulated to avoid sugar accumulation and chip darkening upon frying. The threshold of ethylene concentration affecting fry darkening is lower than 0.4 μl L^-1 ^and is saturated at 4 μl L^-1 ^[[85], and references therein]. These levels are similar to those that trigger respiratory stimulation [0.02 μl L^-1 ^for partial effects and 2 μl L^-1 ^for full stimulation, respectively [86,87]].

Much less information is available on potato endogenous ethylene. It is produced at low rates in unchallenged tubers, but in wound-induced tubers hormone levels inducing respiratory responses may be reached, and thus potato tuber ethylene responsiveness may represent a physiological response to wounding [86]. Indeed, levels of endogenous ethylene up to 0.4 μl Kg^-1 ^h^-1 ^upon environmental stress have been reported [[88,89] and references therein] suggesting that in appropriate post-harvest settings chilling ethylene may play a role in CIS.

In agreement with these arguments, 1-methyl cyclopropene (1-MCP), an ethylene competitive inhibitor, counteracts chip darkening induced by exogenous ethylene [[90-92] and references therein].

It thus appears that many phenomena associated with cold sweetening may be accounted for an upsurge in endogenous ethylene production. Intriguingly, both ethylene-responsive (EREBP or ERF) and cold-responsive (DREB/CBF) transcription factors belong to the same AP2/ERF family of transcription factors [93]. Some members of this family can bind to both cis-elements (GCC and CRT/DRE, respectively) albeit with different affinities, as in the case of the tobacco ethylene-inducible Tsi1 transcription factor. Tsi1 over-expression results in enhanced expression of biotic, GCC-controlled pathogenesis-related genes as well as abiotic stress signalling (CRT/DRE-controlled) pathways [94]. It is interesting that the tomato counterpart of Tsi1 (Pti6) and its closely matching potato counterpart showed enhanced expression in our dataset (Les.3574.1.S1_at; TA30534_4113; 4.65-fold). A similar case is the abiotic Ethylene-Responsive Factor Like Protein 1 in hot pepper [CaERFLP1; [95]]. Again, our dataset indicated enhanced expression of this factor (Les.4102.1.S1_at; TA24509_4113; 3.58-fold). In *Arabidopsis*, a recent report indicates that TINY, a DREB-like factor strongly activated by drought, cold and ethylene, is capable of binding both CRT/DRE and GCC elements with similar affinity. In the same report, ethylene and cold were shown to up-regulate CRT/DRE- and GCC-controlled genes, respectively [96]. Numerous, similar cases of transcription factors with such potential cross-functionality are emerging especially in the Solanaceous species [97-100].

Overall, based on our own data, TED database and numerous further reports [[68,70,101], and references therein] cold and ethylene stimuli appear to cause similar effects on potato tubers such as sugar accumulation, heat shock responses, flavonoid and carotenoid accumulation and enhanced (cyanide-insensitive) respiration. At a gene level, "Cold-Induced 7" protein, acid invertase, enolase, alternative oxidase, ELIP and Glu-6-P-dehydrogenase, to mention only a few among the best-known entries in Table 6, are induced by both triggers.

## Conclusion

Our tomato-potato heterologous approach proved to be essential in planning qPCR experiments regarding cold-responsive gene family members and hinted at further cold-triggered processes that accompany CIS. This suggests that the wealth of available sequence information provided by current plant databases can be fruitfully exploited using heterologous GeneChip approaches for a wide range of species. This applies at least for preliminary screenings that aim to identify candidate genes for further study using more precise techniques such as qPCR. The assessment of alignment quality using a global match procedure should lead to the identification of subsets of highly conserved genes for virtually all species with a reasonable level of phylogenetic relatedness to a "GeneChip available" species. As a result, expression data for genes whose sequence information is available can be validated and optimized while still providing an insight into overall expression trends for all the genes represented in the GeneChip. The potentially low number of sequences above a desired high-confidence threshold in poorly related species is in any case likely to greatly exceed the number of genes that can be treated using traditional profiling approaches. Furthermore, the use of a ready, highly standardized platform with publicly available probeset data such as the commercially available GeneChip should reduce cross-laboratory differences, which may affect custom array approaches.

The GeneChip-assisted qPCR dataset provides a unifying picture of transcriptional events during the first 26 days of CIS. This should prove helpful when trying to ascertain the contribution of various carbohydrate-associated genes to CIS.

In addition to detecting a previously unknown early burst of expression of several carbohydrate-associated genes, our data highlight the key role of β-amylases, which undergo an early enhanced expression. In particular, we identified a β-amylase (*St-BMY7*) at the sequence level that shows maximum homology to a Trx-regulated *Arabidopsis *counterpart, *BMY7*. This, coupled with the activation of redox machinery, suggests that thiol signalling may play a critical role in early cold-sweetening events, especially in the breakdown of starch. In fact, since starch synthesis is known to be modulated by thiol signalling [102], further issues related to the metabolism of starch may be controlled by redox cues.

At least for the subset of early cold-responsive gene family members represented in the GeneChip, no phosphorolytic starch degradation can be seen from our transcriptional data, while an unexpected accumulation of SuSy transcript is evident in the first days. In accordance with previous investigations, transcript profiling supports a continuative role of SPS and acid invertase in CIS.

An intricate crosstalk of regulatory molecules including ethylene and sugars appears to be triggered within a few days at the onset of cold incubation in potato tubers. Ethylene production in potato tubers may be a specific response to chilling or a vestigial response due to relatedness to climateric Solanaceous species such as tomato. Further research is needed to assess the contributory/causative role of ethylene in CIS. It would also be interesting to determine to what extent the manipulation of endogenous, cold-induced ethylene in potato tubers could positively impact CIS.

The identification at a sequence level of various enzymes well established as playing a role in CIS and the discovery of several unexpected CIS-associated global expression trends provides new molecular and conceptual tools for further understanding this phenomenon. This would then improve our knowledge of key food safety issues such as potato chip darkening and acrylamide content upon frying.

## Methods

### Plant material

Field-grown potato tubers (*Solanum tuberosum *cv Hermes) were harvested at physiological maturity (as judged by skin setting) and subjected to a two-week curing period (25°C) before storage at 17°C in the dark for one month. Tubers devoid of defects and of a similar size were incubated at 4°C in the dark in an aerated chamber (90% relative humidity). For the GeneChip experiments, two biological replicates (two distinct tubers) for both control (17°C) and cold-incubated tubers (4 days at 4°C) were used. Tuber material was collected by punching the pith with a 15-mm cork-borer taking care to avoid any vascular tissue. Disks of about 0.5 cm were immediately sliced in liquid nitrogen and stored at -80°C until use.

### Sugar determinations

Tuber disks were ground to a fine powder in liquid nitrogen. For each tuber, 500 milligrams were resuspended in 5 ml of 80% (v/v) ethanol and incubated in a shaking water bath at 75°C for 1 hour. Two milliliters of the resulting suspension were centrifuged at 15,000 *g *and 7 μl of supernatant were used for triplicate determinations. Sucrose, glucose and fructose were determined enzymatically using a UV-method (Boehringer Manheim/R-Biopharm Cat. Nr. 716260035) according to the manufacturer's instructions. A Tecan spectra plate reader equipped with a 340 nm filter was used for absorbance readings. The automated readings were carried out using Magellan software. For each sampling time during sweetening, three independent tubers were assayed and results are reported as means ± SD.

### RNA extraction

A method based on a Qiagen RNeasy plant mini kit was used with slight modifications. Tuber disks (see above) were ground to a fine powder in liquid nitrogen and 1 g of powder was routinely used. To avoid starch interference and improve the RNA yield, 1 vol of Qiagen RLT extraction buffer was combined with an equal volume of the following solution: 2 % (w/v) CTAB (Cetyl Trimethyl Ammonium Bromide); 2% (w/v) PVP 40; EDTA 25 mM; Tris/HCl 100 mM (pH 8). The resulting milky mixture (buffer A) was brought to 2 M NaCl and beta-Mercaptoethanol was added (1% final concentration). For each gram of tuber powder, 1.5 ml of buffer A was used. Subsequent steps in RNA preparation were carried out according to the manufacturer's instructions, the only exception being that, when spin column capacity was exceeded, washes were repeated when necessary to allow for the higher volumes.

### qPCR experiments

Total RNA, extracted as above, was subjected to an extensive DNase treatment using a TURBO DNA-free kit (Ambion). Five micrograms of each sample were reverse transcribed into cDNA using a high-capacity cDNA archive kit (Applied Biosystems). qPCR amplification was carried out using an ABI Prism 7000 sequence detection system (Applied Biosystems) in accordance with the default ABI Prism 7000 PCR program for PCR conditions. Potato EF1-alpha (AB061263) was used as an endogenous control since it is an appropriate reference for cold stress in potatoes [108]. Gene-specific primers/TaqMan probes were used. When the exon-intron structure of a potato gene was known, TaqMan probes were designed over an exon-intron boundary. Probe and primer sequences are reported in Additional file 4. PCR reactions were carried out using 50 ng of cDNA and TaqMan Universal PCR master mix (Applied Biosystems), following the manufacturer's instructions. Relative quantitation of each individual gene expression was performed using a comparative CT method, as described in the ABI PRISM 7700 Sequence Detection System User Bulletin #2 (Applied Biosystems). Data points in qPCR time courses are reported as means ± SD of two technical replicates of a single, representative profile.

### Microarray Experiments and Statistical Analysis

Two biological replicates were used by extracting total RNA from two distinct tubers for both the control (17°C) and cold treatment (4 days at 4°C). RNA quality was assessed by agarose gel electrophoresis and spectrophotometry. RNA processing for use on the tomato GeneChip Array was carried out as in [34]. Hybridization, washing, staining, and scanning procedures were performed as described in the Affymetrix technical manual.

Microarray analysis was performed using R/Bioconductor [109]. Expression measures for each probeset were obtained using GCRMA [110], which is a multi-array analysis method estimating probeset signals considering the physical affinities between probes and targets. Normalization was performed using a quantiles method [111]. To discard probesets with the lowest variability from the two experimental conditions (control vs. cold treatment), thus reducing the number of non-informative genes, a filter based on an interquartile range was applied (IQR = 0.18).

This filtering method discards approximately half of the total amount of probesets. To identify a statistically reliable number of differentially expressed genes from the two conditions, a linear model was performed [112]. To assess the differential expression, an empirical Bayesian method

[113] was used to moderate the standard error of the estimated log-fold changes. This method, applying a hierarchical model, allows to estimate the hyperparameters of prior distribution directly from the data. The advantage is an improvement in error estimates for genes with a small number of replicates. To control the number of false positives, a Benjamini-Hochberg multiple test correction of the false discovery rate [114] was applied (adjusted P-value <= 0.05). This procedure led to the final number of 1,854 differentially expressed probesets.

We used a hierarchical clustering method to concisely represent the expression profiles of all the 1,854 differentially expressed probesets, and the intersection between these probesets and those selected by matching tomato vs. potato at a 70% perfect alignment (%_PERF_ALIGN) threshold. The length of each branch of the dendrogram indicates (1 – Pearson) correlation coefficients as a measure of similarity. The resulting tree structure shows the relationship between the expression profiles of each probeset. The figure is a heat map of the 1,854 differentially expressed probesets. The expression values of each probeset are divided by the median value, and the log_2 _of this ratio is reported. Red indicates samples where the probeset is more expressed, blue where the expression is smaller. Probesets (rows) are reordered based on their similarity, as indicated in the respective dendrograms. The column between the dendrogram and the heatmap represents the probesets either differentially expressed and selected with the 70% perfect alignment threshold (yellow), or the probesets differentially expressed but not selected by the Perfect Alignment threshold (black).

### Tomato vs. potato global matching

A BLAST [115] of all the tomato targets – derived from Affymetrix's Tomato GeneChip [116] – against the total amount of TIGR 's Potato EST Release 2 [117] was performed. Standalone BLAST was run locally using default settings. In order to get a customized BLAST, a local potato database was built by downloading TIGR TA Potato ESTs (Release 2) and formatting them with the BLAST formatDB integrated function. Finally, a global query for all the tomato targets was performed. Results were parsed to colligate the BLAST indexes into a single file, which enabled them to be easily compared and further indexes defined. A BLAST search was executed on an Intel Pentium IV with a 3 Gb RAM running Linux. A redundant set of 430,000 alignments was obtained, since the tomato target sequences frequently produced numerous multiple matches of various scores, unless a threshold value for alignment quality/magnitude was set. By filtering matches below a 5% %_PERF_ALIGN threshold (Table 1; and for more details see below), we reduced the number of entries to 269,474.

To characterize the alignment quality and reduce the number of redundant alignments with low matching scores, three different quality indexes were defined and added to the global match file as follows:

- %_ALIGN/TARG: Rough ratio between aligned portion of a potato's EST against the total length of a tomato's target (ALIGN_LENGTH/LENGTH_TOM) * 100 to roughly assess alignment regions. Scores higher than 100% mean that there were some 'holes' in the alignment.

- %_PERF_ALIGN: Corrected ratio between aligned portion of a potato's EST against the tomato's target (%_ALIGN/TARG * %_IDENTITY)/100. This correction is needed to obtain the percentage of net alignment, thus hiding the effects of non-contiguous alignment regions. Scores of 100% meant that the entire length of the tomato target sequence was aligned. Matches below the 5% PERF_ALIGN threshold were excluded.

- %_STOP_DIST: Ratio between position of last nucleotide aligned in a tomato's target against the total length of the tomato's target (LAST_POS_TOM/LENGTH_TOM) * 100. As Affymetrix target region design procedure is oriented towards 3' end of tomato mRNAs, this index may help when trying to predict whether a lack of alignment involves coding sequences or more downstream regions in the 3' UTR.

## Authors' contributions

PB and PP designed and planned the experiments. PB and AM conducted the RNA preparation, qPCR and sugar determinations. OB and FV carried out the bioinformatics procedures. PB drafted the manuscript. PP corrected the draft. PR conceived the study, participated in its design and coordination and finalized the written manuscript. All authors read and approved the final manuscript.

## Supplementary Material

Additional file 1 **Tomato-Potato Global Match File part 1 (GMF 1)**. All GeneChip tomato target sequences were blasted against TIGR potato TA (release 2). Matches below a 5% perfect alignment threshold were discarded. The alignments are listed in separate sheets due to size constraints and are split into five parts (part 1 to 5). Parts 1 and 2 are contained in this first file (GMF 1), and parts 3 to 5 are included in additional file 2 (GMF 2). The first part of the Global Match File also includes alignments scoring above the 90% and 70%_PERF_ALIGN thresholds (ALL_90% and ALL_70% sheets, respectively). As a single probeset can produce more than one hit, both > 90% and > 70% sheets are accompanied by a further sheet listing non-redundant probesets above the corresponding threshold. The Global Match File was split into two parts due to upload size limits. Ideally the two files should be merged back together, this would then enable searches to be performed with just one query across the whole Global Match File alignments Click here for file

Additional file 2 **Tomato-Potato Global Match File part 2 (GMF 2)**. Global Match File parts 3 to 5; see additional file 1. Click here for file

Additional file 3 **differentially expressed genes (DEG) list**. DEG are sorted in order of decreasing fold induction (expressed as Log fold changes). Each tomato probeset is accompanied by the three best matching potato TA sorted in order of decreasing alignment (%_PERF_ALIGN). For each TA, perfect alignment values, TA identification number and TA annotation are reported. Fields are separated by the "-|-" string. Click here for file

Additional file 4 **List of primers and TaqMan probes used in qPCR analysis**. Sequences of Gene-specific primers and TaqMan probes used for qPCR experiments are reported. Click here for file
