# Supplementary material for: Heterologous microarray experiments allow the identification of the early events associated with potato tuber cold sweetening
Source: BMC Genomics. 2008 Apr 16;9:176. doi: 10.1186/1471-2164-9-176 (PMC2358903; doi:10.1186/1471-2164-9-176)
Supplement: Additional file 4 — List of primers and TaqMan probes used in qPCR analysis. Sequences of Gene-specific primers and TaqMan probes used for qPCR experiments are reported. [file 1471-2164-9-176-S4.doc]

| *Potato TA/singleton* *(Best matching tomato probeset)* | *Annotation* | *Description* | *5’-3’ sequence* |
| --- | --- | --- | --- |
| TA25853_4113  (Les.3195.1.S1_at; Les.3195.2.S1_at) | GWD  chloroplast precursor | primer forward | CATTCAGGTGCAGATCTTGAGTCT |
|  |  | primer reverse | GGACTCCAACCATAAAGCCTTCTC |
|  |  | TaqMan probe | ATGGGCTACAAAACTG |
| CV475083  (Les.3551.1.S1_at) | ER24 | primer forward | GCTGGAGCACAAGTTCAAACG |
|  |  | primer reverse | CGCCGCCTTCTTGTTCAATC |
|  |  | TaqMan probe | CACCGTCGAATTTC |
| TA42474_4113  (LesAffx.37983.1.S1_at) | Neutral invertase | primer forward | AAGCACGATACAGAAGGGAAAGAAT |
|  |  | primer reverse | GAGCCAAAAATAACTTCGCATGTGA |
|  |  | TaqMan probe | ATGCAGGCGCTTCACT |
| TA24089_4113  (Les.2820.1.S1_at;  Les.2820.2.S1_at ) | Glucan Phosphorylase | primer forward | GGATTGGGTCCTGAAAACTGAAAAG |
|  |  | primer reverse | TGCTTCCCTCCACTCATTTTGAA |
|  |  | TaqMan probe | CAGAATTGCAGAAGTTTG |
| TA33682_4113  (LesAffx.53231.1.S1_at) | PCT-BMYI | primer forward | GCTACTGGAGCATGGTGACA |
|  |  | primer reverse | GCCCCAGTTCCTTGGTATATACTTT |
|  |  | TaqMan probe | TCCTGCTGCTACTATTC |
| TA25537_4113  (Les.2560.1.S1_at) | ACO1 | primer forward | CGAATACAGAGAGGTAATGAGGGATT |
|  |  | primer reverse | AGTCCAAGATTTTCACATAGCAAGTCA |
|  |  | TaqMan probe | CCTCTGCCAATTTC |
| TA24067_4113  (Les.157.1.S1_at) | SuSy | primer forward | CTTTCGGTCTGACTGTTGTTGAG |
|  |  | primer reverse | TGGACCACCTTGATTTGTTGCA |
|  |  | TaqMan probe | CAAACCGCAGCTCATG |
| TA23155_4113  ( Les.2844.1.S1_at) | P-BMY7 | primer forward | GCAGCCACAAGATGCACTATG |
|  |  | primer reverse | TGAGCTTCCTGAGTTGCTAATGC |
|  |  | TaqMan probe | CTTGCCTAACCAACTTC |
| TA24502_4113  (Les.3208.1.S1_at) | UGPase | primer forward | CCATCGAGTTGGGACCTGAA |
|  |  | primer reverse | GGGAATAGACTTGAAACGGCCTAAG |
|  |  | TaqMan probe | AAGTTGGCCACCTTCTTGAA |
| TA26174_4113  (Les.3522.1.S1_at) | SPS | primer forward | GAAGACAATTCAGCTGACTACTGCTA |
|  |  | primer reverse | GTGCCTGAATTCGCATTACTTTTCT |
|  |  | TaqMan probe | CTGGGACGGTTCCTC |
| AB061263  Potato EF1-alpha | EF1-alfa | primer forward | GGTGATGCTGGTATGGTTAAGATGA |
|  |  | primer reverse | CAGCAAAACGACCCAATGGT |
|  |  | TaqMan probe | ATGGTTGTTGAGACCTTTG |
| TA26908_4113  (Les.2702.1.S1_a_at) | Acid invertase | primer forward | GGTGTCGTTGTAATTGCTGATCAAA |
|  |  | primer reverse | CTCGACCATCAGCTCCTTTAGAAAT |
|  |  | TaqMan probe | CTAACGCCAGTTTACTTCTAC |

Supplemental Table S3.

List of primers and Taqman probes used in Real Time PCR analysis.
